# Phytolith assemblage analysis for the identification of rice paddy

**DOI:** 10.1038/s41598-018-29172-5

**Published:** 2018-07-19

**Authors:** Xiujia Huan, Houyuan Lu, Jianping Zhang, Can Wang

**Affiliations:** 1 Institute of Geology and Geophysics, Chinese Academy of Sciences, Key Laboratory of Cenozoic Geology and Environment, Beijing, 100029 China; 2 University of Chinese Academy of Sciences, Beijing, 100049 China; 3 CAS Center for Excellence in Tibetan Plateau Earth Sciences, Beijing, 100101 China; 4 Hebei Normal University, School of history and culture, Shijiazhuang, 050024 China

## Abstract

The rice arable system is of importance to both society and the environment. The emergence of rice paddies was a crucial step in the transition from pre-domestic cultivation to systematic land use and management. However, many aspects of the formation of rice farming systems remain unclear. An important reason is the lack of reliable methods for identifying early rice paddies. One possible means of remedying this knowledge deficit is through analysis of phytolith assemblages, which are closely related to their parent plant communities. In this study, phytolith assemblages from 27 surface soil samples from wild rice fields, 91 surface soil samples from modern rice paddies, and 50 soil samples from non-rice fields were analysed to establish a discriminant function. This discriminant function enabled classification of 89.3% of the samples into appropriate groups. Further, the results suggested that phytolith assemblages can be used to identify rice fields and differentiate between wild rice fields and domesticated rice fields. The method was demonstrated to be an effective way of utilising the large amounts of unidentifiable phytoliths discovered at archaeological sites to provide a modern analogue that may be a valuable key to unlocking the past.

## Introduction

Rice paddies are crucial for global food security because rice is the staple food of nearly half the world population^1^. Eastern Asia, Southern Asia, and South-Eastern Asia account for almost 90% of rice production and 88% of the rice cultivation area^2^. The history of Asian societies, human populations and the evolutionary history of rice as a crop are inseparable^3^; rice production was a prerequisite for the rise of civilization in monsoon Asia. The earliest confirmed rice paddy cultivation began in the coastal wetlands of Eastern China about 7,700 cal yr B.P.^4^; however, phytolith evidence showed that rice domestication might have begun as early as 9,400 cal yr B.P. in the lower Yangtze Basin^5,6^. Exploring the transformation from pre-domestic cultivation to current systematic, large-scale land use and management is the key to understanding the formation of rice farming systems; however, when, where, and how rice paddies emerged remains unclear, largely because of the lack of reliable methods to identify early rice paddies.

Currently, the methods for studying ancient and present-day rice paddies mainly focus on the physical and chemical properties of paddy soil^7,8^, such as micromorphological features^9^, organic matter^10–12^ (including organic carbon^13–18^ and nitrogen^19,20^, fatty acids and polycyclic aromatic hydrocarbons^21–26^), forms of iron^27,28^, mineral characteristics^29^, bacterial communities^30–34^, soil fertility^35^, pollen features^36^, and phytolith accumulation^37^. Although these methods are effective for analysing the characteristics of paddy fields, they can only be applied to known paddy fields.

Phytoliths are siliceous bodies present in certain plant tissues; plants absorb silica in a soluble state from groundwater and accumulate solid silica in both intracellular and extracellular locations^38^. Phytoliths can be used to identify particular genera or species based on shape, size, and other anatomical features^38–40^. Rice bulliform phytoliths^41,42^ and double-peak phytoliths^43,44^, which differ between wild and domesticated rice, have been employed to study the origin and domestication of rice^5,6,45^. Fujiwara *et al*.^46^ established the paddy identification standard as 5000 rice bulliform phytoliths in 1 g of dried soil sample and this standard has been applied to identify Neolithic paddies in the Shandong and Zhejiang Provinces^47,48^. However, careful consideration is required when applying this standard because if the crop is harvested by uprooting or basal cutting, only a few bulliform phytoliths will remain in the field^49,50^.

Notably, a rice system does not contain paddy soil alone; it is a relatively independent ecosystem including other plant species that might be of use for identification. Rice arable systems are of importance to both the society^3,51–54^ and the environment^55–57^. As rice arable systems are associated with specific grasses, weed assemblages^58–61^ can be used to determine changes in arable systems. Recently, phytolith assemblages, based on the proportion of fixed and sensitive types have been used to distinguish between wet and dry cultivation systems^62–64^; however, similar studies using phytolith assemblages to differentiate wild and domesticated rice paddies are rare.

**Figure 1:**
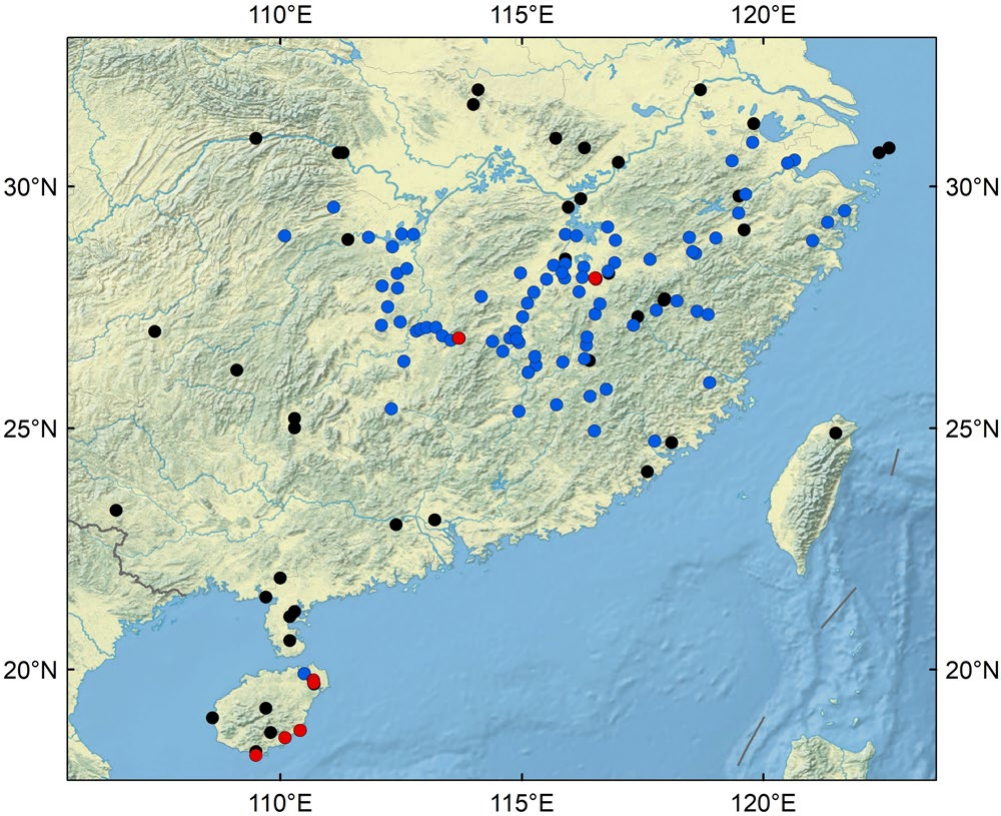
Geographic locations of samples. Red dots represent soil samples from wild rice fields; blue dots represent soil samples from domesticated rice paddies; black dots represent soil samples from non-rice fields. (The figure was generated using GRASS GIS 7.2.1: GRASS Development Team, 2017. Geographic Resources Analysis Support System (GRASS) Software, Version 7.2. Open Source Geospatial Foundation. Electronic document: http://grass.osgeo.org).

In the present study, a detailed discriminant analysis was conducted on modern soil samples (Fig. 1) to develop a method for identifying rice fields using phytolith assemblages as indicators. The results demonstrated that modern phytolith assemblages can be used to differentiate between wild rice fields, domesticated rice paddies, and non-rice fields.

## Results

### Phytolith assemblages in selected samples

**Figure 2:**
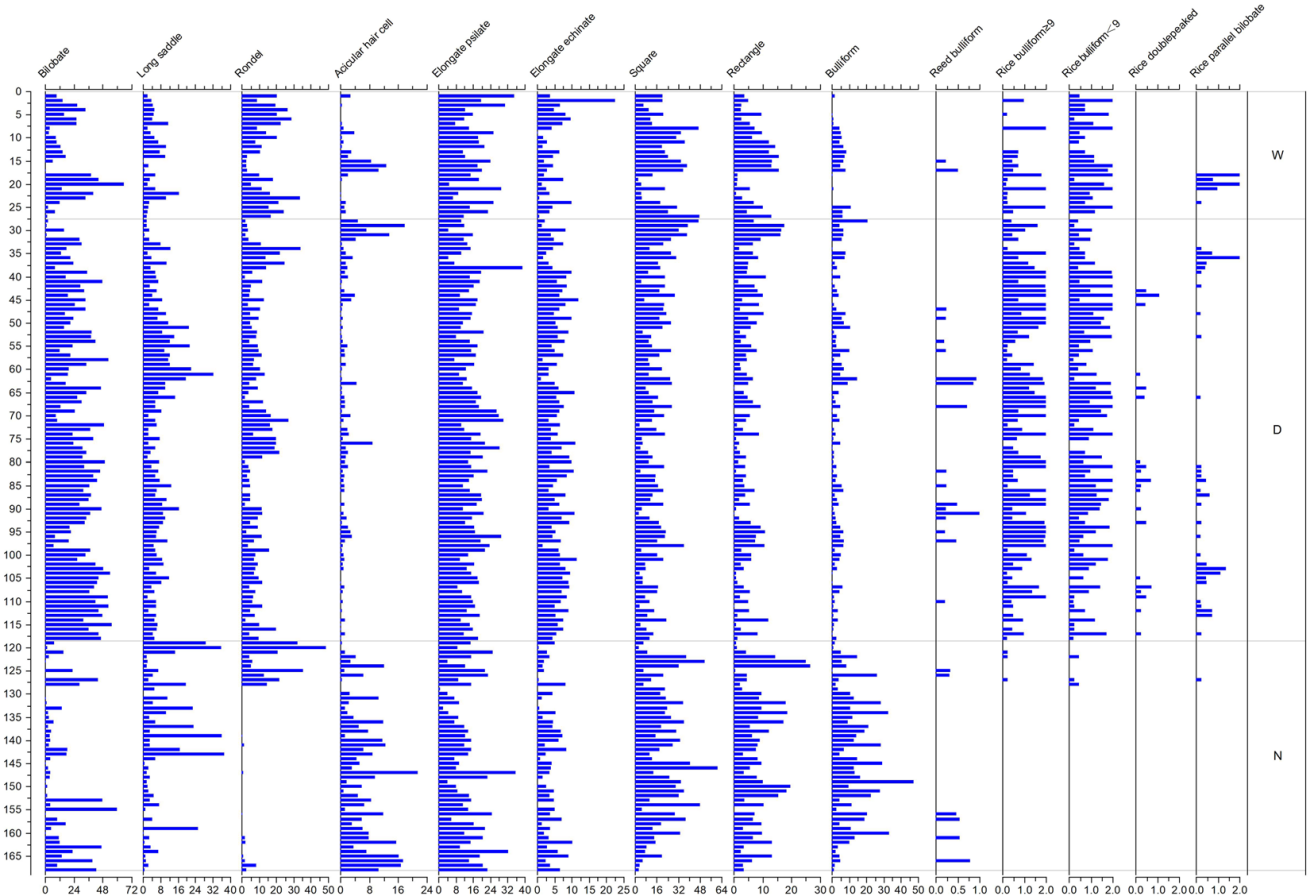
Percentage diagram of the major phytolith morpho-types in selected samples. Group code on the right: W represents samples from wild rice fields; D represents samples from domesticated rice paddies; N represent soil samples from non-rice fields. For sample codes, refer Supplementary Table S2.

**Figure 3:**
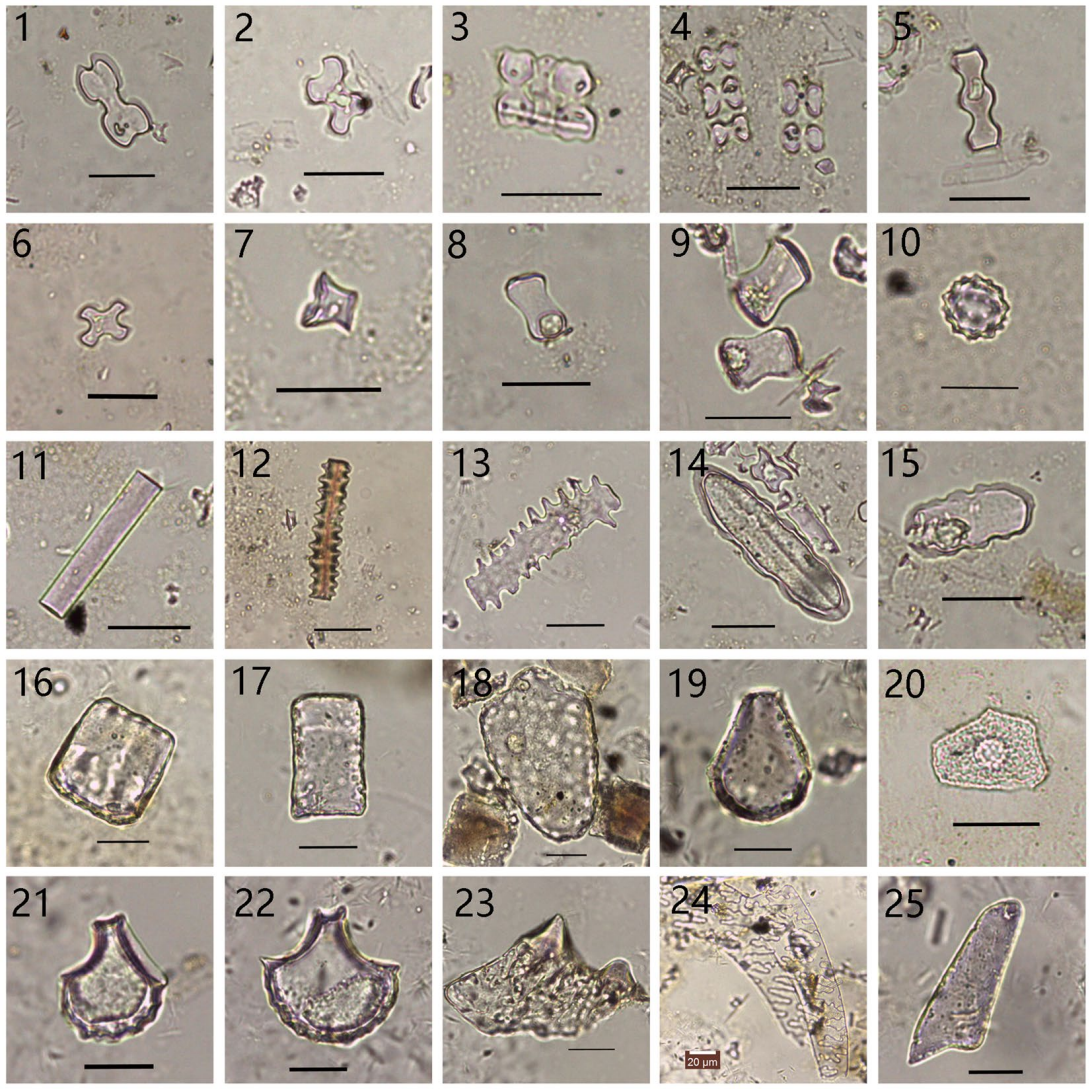
Major phytolith morpho-types in rice paddy soil 1–2: Bilobate; 3–4: Parallel-bilobate 5: Cylindrical polylobate; 6: Cross; 7: Rondel; 8–9: Long saddle; 10: Globular echinate; 11: Elongate psilate; 12–13: Elongate echinate; 14–15: Trapeziform sinuate; 16: Square; 17: Rectangle; 18: Reed bulliform; 19: Bulliform; 20: Cyperaceae; 21: Rice bulliform with < 9 fish-scale decorations; 22: Rice bulliform with ≥ 9 fish-scale decorations; 23: Rice double-peaked; 24: Barnyard grass husk; 25: Acicular hair cell (Scale bar 20 μm).

A total of 170 soil samples were collected and 168 of these contained abundant phytoliths. Twenty-one phytolith morphotypes were identified (Fig. 2), including bilobate, cylindrical polylobate, cross, long saddle, short saddle, rondel, trapeziform sinuate, acicular hair cell, elongate psilate, elongate echinate, square, rectangle, bulliform, Cyperaceae-type, globular echinate and barnyard grass husk-type. Soil samples from wild rice and domesticated rice fields were characterised by the presence of bulliform phytoliths with fish-scale decorations. Characteristic phytolith types are shown in Fig. 3.

All surface soil samples from the wild rice fields were characterised by high rates of square (22.17%), bilobate (18.19%) and elongate psilate (17.62%) phytoliths. Furthermore, the proportion of bulliform phytoliths with ≥9 fish-scale decorations was 0.55%, whereas that for bulliform phytoliths with <9 fish-scale decorations was 1.55%. The average phytolith concentration in these samples was ~1.18 million particles/g; the highest phytolith concentration was 6.34 million particles/g (WN-BT8), whereas the lowest concentration was 5,200 particles/g (WN-BT6).

Among 93 soil samples from domesticated rice paddies, no phytoliths were observed in samples DTZJ9 and DTZJ11. Phytolith assemblages from the other 91 domesticated rice paddy samples were characterised by fairly high rates of bilobate (30.54%), square (15.96%), and elongate psilate (15.90%) phytoliths. The proportion of bulliform phytoliths with ≥9 fish-scale decorations was 1.43%, whereas that for bulliform phytoliths with <9 fish-scale decorations was 1.14%. The average phytolith concentration in these samples was ~3.03 million particles/g. The highest concentration was 6.61 million particles/g (HL-OS1), whereas the lowest concentration was 15800 particles/g (DTZJ3).

Phytolith assemblages from the 50 non-rice field samples were different from those of rice field samples in that high rates of square (22.14%), elongate psilate (13.67%), bulliform (12.78%) and bilobate (11.47%) phytoliths were present. In particular, four samples (10CL-FB1, 10CL-FB2, WN-BT5, and ZX-4) in this group contained a low rate of rice bulliform phytolith particles. Although the four soil samples did not come from wild rice or domesticated rice fields, their sampling locations were extremely close to wild or domesticated rice fields. Therefore, it is possible that these samples contained rice bulliform phytoliths because of soil tillage or other soil-disturbing activities.

### Numerical analysis of modern phytolith data

**Figure 4:**
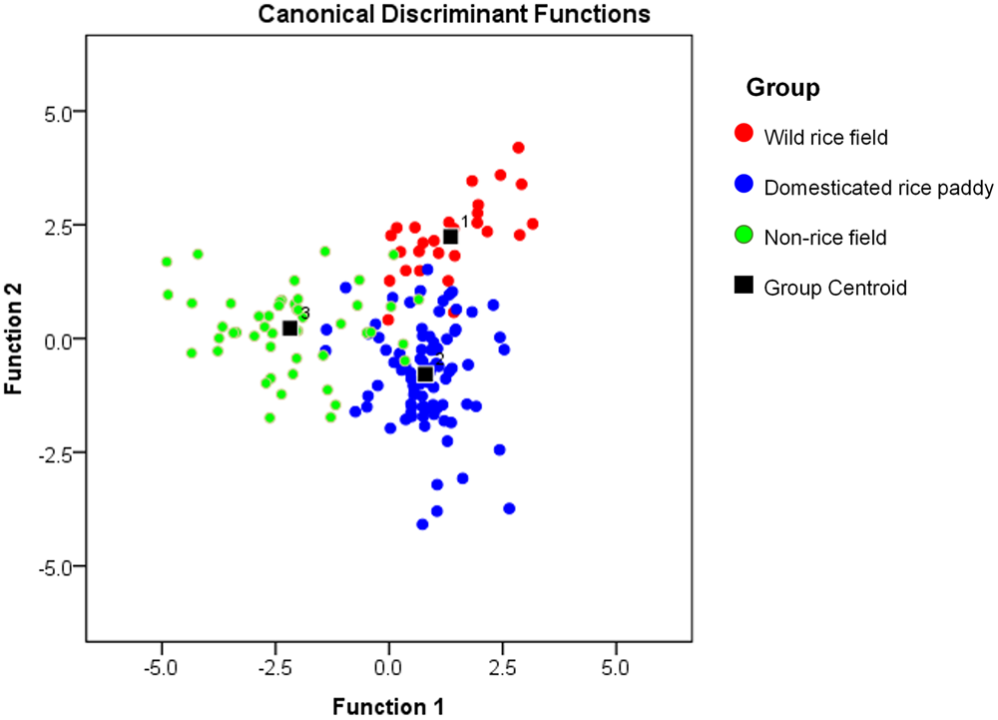
One hundred and sixty-eight samples plotted against the canonical discriminant functions 1 and 2 and their group centroids corresponding to three groups.

We used the data on 21 phytolith types (Supplementary Table S2) in a discriminant analysis. The discriminant scores of 168 samples were plotted along the first two discriminant functions, accounting for 64% and 36% of variance, respectively (Fig. 4). The results showed that the three group centroids were clearly separated, marginally overlapping with each other (Fig. 4; black square).

The results of the discriminant analysis showed that 89.3% (150 out of 168) of the original grouped cases were classified correctly (Table 1). Only two of the 27 samples from the wild rice field were misclassified. Of the samples from the domesticated rice paddies, 90.1% were classified correctly and nine samples were misclassified. Seven samples in the non-rice field group were classified in the rice-field group. Regarding cross-validation (each case was classified using the functions derived from all the other cases), 82.1% of the cross-validated grouped cases were classified correctly (Table 1). The high degree of correct classification suggested that the classification of the surface soil samples into the three groups was statistically robust.

**Table 1:** Classification results of discriminant analysis of 168 samples.

Group^a^			Predicted Group Membership			Total
			1	2	3	
Original^**b**^	Count	1	25	2	0	27
		2	6	82	3	91
		3	2	5	43	50
	%	1	92.6	7.4	0.0	100.0
		2	6.6	90.1	3.3	100.0
		3	4.0	10.0	86.0	100.0
Cross-validated^**c, d**^	Count	1	24	3	0	27
		2	11	74	6	91
		3	2	8	40	50
	%	1	88.9	11.1	0.0	100.0
		2	12.1	81.3	6.6	100.0
		3	4.0	16.0	80.0	100.0

^a^Group 1: Wild rice group; Group 2: Domesticated rice group; Group 3: Non-rice group.

^b^89.3% of original grouped cases correctly classified.

^c^Cross-validation was performed only for those cases in the analysis. In cross-validation, each case was classified by the function derived from all other cases.

^d^82.1% of cross-validated grouped cases correctly classified.

## Discussion and Conclusions

Earlier studies on phytolith assemblage focused mainly on palaeoenvironment reconstruction^65,66^. Only a few specific crop-related phytoliths are highly valued for their application in archaeology^41,43,67–71^. Therefore, a large proportion of unidentifiable phytoliths discovered at archaeological sites are undervalued. Excluding a few cases^62–64^, phytoliths could be exploited differently for archaeological applications by combining with statistical analyses, such as correspondence analysis, discriminant analysis, and principal component analysis.

This study demonstrated that rice paddies could be distinguished by their phytolith assemblages. Soil samples from both the wild rice fields and domesticated rice paddies were characterised by high proportions of bilobate, square and elongate psilate phytoliths, whereas the non-rice field samples exhibited diverse phytolith assemblages dominated by the rondel type. Furthermore, according to phytolith grouping for fixed and sensitive types^64^, a higher proportion of bilobate phytoliths (a fixed type) was observed in the soil samples from the domesticated rice paddies, whereas a higher rate of elongate psilate (a sensitive type) phytoliths was obtained from the wild rice fields. This probably indicates that wild rice habitats are swampier than those of domesticated rice. The proportion of rice bulliform phytoliths is informative, although it is relatively low. Rice bulliform phytoliths are unique to rice plants and the decorations on the edges of rice bulliform phytoliths can be used to differentiate wild rice from domesticated rice^41,42^. However, because of the limited number of samples from Southern China, the results were not compared with samples from Northern China, South Asia, and Southeast Asia, which are also major rice-producing areas.

This study analysed the phytoliths present in 168 soil samples collected from wild and domesticated rice fields and non-rice fields to establish a discriminant function, and demonstrated that these phytolith assemblages correctly classified 89.3% of the samples. The results statistically support the development of a robust method for identifying rice paddies and distinguishing between wild and domesticated rice fields. The method was demonstrated to be an effective way of utilising the large numbers of unidentifiable phytoliths from archaeological sites and this modern analogue should provide a valuable key to unlocking the past.

## Methods

In this study, a total of 170 soil samples were collected for phytolith analysis (Fig. 1). These included (1) 27 surface soil samples from wild rice fields in Jiangxi, Hu’nan and Hainan, (2) 93 surface soil samples from domesticated rice paddy fields in Zhejiang, Jiangxi, Hu’nan, Fujian and Hainan, and (3) 50 soil samples from non-rice fields in Southern China (40 of these were selected from the modern phytolith database for Chinese surface soil^65^). The geographic distribution of rice fields is 18.23°N–30.91°N and 109.5°E–121.33°E, so we chose non-rice fields samples located at 18.3°N–32°N and 106.6°E–122.6°E to ensure all studied samples came from similar climatic regions. Surface soils with a depth of 0–5 cm (in most cases from the top 5 cm, but sometimes from the top 3 cm) were collected and mixed evenly before analysis. Leaves, roots, and plant litter were removed prior to sampling. Details of all the samples are presented in Supplementary Table S1. All necessary permits for sample collection from Jiangxi were obtained from the Jiangxi Academy of Agricultural Sciences. No permit was required to collect samples from the other locations.

Phytoliths were extracted from the soil samples according to the procedure described by Zhang^72^, with minor modifications. Initially, 5 g of soil samples was weighed. Subsequently, 30% H_2_O_2_ and 15% HCl were added to the samples to remove organic matter and carbonates. The samples were then subjected to heavy liquid flotation using ZnBr_2_ (density, 2.35 g/cm^3^) to separate the phytoliths, which were subsequently mounted on a slide with Canada Balsam. The phytoliths were counted and identified under a Leica microscope at 400X magnification. More than 400 phytolith particles from grass were counted in each sample. A few woody phytoliths were identified in this study; however, because these can be highly influenced by local environment and climatic factors, they were excluded to minimize possible bias^73^. Identification of phytoliths was performed using previous studies as references^39,41,67,74^.

Discriminant analysis was performed using SPSS version 22.0. It is a method of statistical inference for deriving linear combinations of variables, called discriminant functions, which are mutually independent^75,76^. These discriminant functions ensure maximum separation among priori sample groups and can also be used to classify new samples with unknown group memberships into one of the priori groups^75–77^. In this study, 168 samples were initially divided into three priori groups (actual); subsequently percentage data of 21 phytolith types were used to establish the discriminant functions and the samples were classified into predicted groups.

## Electronic supplementary material

Dataset 1
